# High tibial osteotomy improves balance control in patients with knee osteoarthritis and a varus deformity

**DOI:** 10.1186/s13018-023-04041-8

**Published:** 2023-07-28

**Authors:** Zheng Zhang, Hai Tao, Yingchun Zhao, Wei Xiang, Hui Cao, Fenghua Tao

**Affiliations:** Department of Orthopedics, Renmin Hospital of Wuhan University, 238, Jiefang Road, Wuchang District, Wuhan, 430060 Hubei China

**Keywords:** Balance control, Osteoarthritis, Knee varus, High tibial osteotomy, Posturography

## Abstract

**Background:**

Impaired knee stability is observed in patients with medial compartment knee osteoarthritis (OA) and varus malalignment. Although high tibial osteotomy (HTO) is widely used to treat OA-related knee varus deformity, its long-term influence on balance control in OA patients is poorly reported. This study aimed to evaluate the impact of HTO on balance control and assess its biological and functional significance.

**Methods:**

Thirty-two patients with medial compartment knee OA as well as varus deformity who were scheduled for HTO underwent static posturographic tests one month pre- and three months as well as one year postoperatively, respectively, along with forty matched control subjects. Radiographic and clinical evaluations were synchronously carried out on patients pre- and postoperatively.

**Results:**

Decreased postural sway was observed in patients one year after HTO. When compared to the control subjects, more postural sway was found in patients one month pre- and three months postoperatively. No difference was observed between the patients and control subjects one year postoperatively. The alignment and joint function of the affected knees significantly improved after HTO.

**Conclusions:**

This study revealed that HTO improves balance control in patients with knee OA and varus deformity. Correct alignment and improved joint function enhance the likelihood of normal postural stability. Hence, this intervention allows the knee joint to recover its corrective compensatory role in postural regulation and should be taken into account for managing knee OA patients.

## Introduction

Knee osteoarthritis (OA) is the most common joint disorder, particularly in elderly people [1]. Patients with medial compartment OA often have a varus deformity due to increased load transmitted through the affected compartment [2–4]. As the disease progresses, patients may experience an inefficient balance control in their daily life due to joint pain, stiffness as well as internal structure changes resulting in somatosensory deficit and performance-based activity limitations [5–7]. Impaired balance control, as mentioned in previous literatures in OA populations, is an important risk factor in the occurrence of fall-related injuries which bring a significant economic burden to society [8–10].

When conservative management fails, surgical interventions are commonly prescribed for treating knee OA and its related varus deformity [11–13]. Lee et al. reported the total knee arthroplasty (TKA) was effective in reducing the postural sway in severe knee OA patients and full recovery of balance control 1 year after surgery [14]. Similar outcome was revealed by Goetz et al. that successful unicondylar knee arthroplasty (UKA) in OA patients can restore postural stability to the level of the contralateral side 16 months after the procedure [15]. Furthermore, improved balance-specific performance for elderly patients after TKA may minimize fall risk and optimize physical functions [16]. However, as a joint-preserving treatment, high tibial osteotomy (HTO) has more advantages over joint replacement in treating knee OA and varus deformity in young and active individuals [17, 18], especially during their postoperative sports activities [19]. In these patients, HTO involves a wedge osteotomy on the medial side of the proximal tibia that orients the mechanical axis of the lower extremity to pass through the ideal position of the tibiofemoral compartment, ensuring the knee joint stability [20, 21]. It was proved that the corrective osteotomy, which is performed in either the femur or tibia or in both bones in patients with knee OA combined with a valgus deformity, is able to improve the kinematics of gait 1 year postoperatively by realigning the weight-bearing lines while maintaining normal knee joint line orientation [22].

Although a small number of literatures have reported a failure to effectively improve single-leg standing stability in normal conditions after HTO [23, 24], studies that comprehensively assess changes in bipedal standing balance control before and after HTO, such as its recovery under available or absent or disturbed visual and proprioceptive conditions, for static balance control that relies on vision and proprioception, remain unclear. Such comprehensive evaluations and long-term follow-ups are important for guiding the clinical decision making of physicians and the postoperative daily activities and rehabilitation of patients.

The primary aim of this study was to compare the changes in balance control in knee varus patients in normal and disturbed conditions before and after HTO. We suggested that these patients may have better postural stability postoperatively due to the corrected lower limb alignment and improved joint function. The secondary aim was to assess the biological and functional significance of the stability restoration after HTO. We also advocated that preoperatively, patients showed less efficient balance control performance compared to the control individuals, which could return to normal postoperatively.

## Materials and methods

### Research design

For evaluating the influence of HTO on balance control, we compared the variations in balance control of knee OA patients pre- and postoperatively by performing posturography in line with the normal scheduled surgical planning. Meanwhile, the same balance control tests were carried out in healthy controls to assess the level of patients’ recovery. Radiographic and clinical evaluations were obtained synchronously with the balance control test. The protocol and design of this observational study were reviewed and approved by the medical ethical committee of Renmin Hospital of Wuhan University. All procedures performed in this study involving human participants were in accordance with the ethical standards of the institutional research committee and with the 1964 Helsinki Declaration and its later amendments. Written informed consent was obtained from each subject before participation.

### Participants

Patients with unilateral medial compartment knee OA and varus deformity were recruited from the outpatient clinic of Renmin Hospital of Wuhan University and scheduled for HTO. Patients were only included if they were diagnosed by the same orthopedic surgeon according to the American College of Rheumatology criteria [25]. Lower extremity alignment and joint space narrowing were assessed from full-length weight-bearing radiographs. If the weight-bearing line was ≤ 35% and the Kellgren and Lawrence radiographic grade was ≥ 3, the patients underwent surgery [26]. Participants were excluded if they had TKA in either of the knees, bilateral knee varus deformity, proven ligament injuries by magnetic resonance imaging (MRI), other musculoskeletal disorders, neurologic impairments, and severe depressive syndromes. Control subjects without lower limb pathology or a knee trauma history were recruited from hospital staff, students, and local communities using advertisements. Finally, 32 OA patients and 40 control subjects, meeting the inclusion criteria, participated in this study.

### High tibial osteotomy

The deformity correction planning was aimed at correcting the lower extremity alignment to a neutral position by HTO. An oblique osteotomy was performed 35mm below the medial tibial articular surface from the cortex to the upper third of the proximal tibiofibular joint. All osteotomies were biplanar opening wedge HTO surgeries and were performed by the same surgeons with standard osteotomy techniques [27, 28]. The preoperative osteotomy angle was measured by a goniometer on coronal and sagittal planes to enable accurate wedge resections. Additionally, the osteotomized gap was gradually opened and filled with three wedged osteotomes, and all osteotomies were fixed with a TomoFix plate (DePuy Synthes, Zuchwil, Switzerland) without bone grafts. A postoperative rehabilitation program was initiated that included straight leg raising, isometric quadriceps, and continuous passive motion the next day postoperatively for two weeks to avoid joint stiffness and muscular atrophy. Moreover, patients used walkers or crutches for a non-weight-bearing period of four weeks and subsequently began a progressive weight-bearing from partial to full over the ensuing eight weeks.

### Posturography

The study patients underwent posturographic tests in a quiet and bright room in the hospital’s inpatient department one month pre- and three months as well as one year postoperatively, respectively. Control subjects were measured three times at the same time and placed as the study patients. All the participants were tested by the same operator on a vertical force platform (Win-Posturo, Medicapteurs, Balma, France). Three strain–gauge force transducers were installed on the platform bottom to sense subject’ body sway according to the displacement of the center of foot pressure (CoP) in a two-dimensional horizontal plane (recording time: 25.6 s, acquisition frequency: 40 Hz). The captured analogue signals are converted to digital forms in the computer. The sway area (in mm^2^) covered by the CoP trajectory was used to quantify postural sway. Lower value of the sway area suggests better balance control precision [29]. Subjects stood bare feet on the platform with a quiet upright position and maintained body stability with their feet abducted at 30^°^, heels apart by 3 cm, and arms along the body. Two visual conditions (eyes open and closed) along with two platform conditions (without and with foam support) were provided during the entire test to imitate different sensory input environments. Posturography performed on firm support with eyes open (C1) or closed (C2) was considered a basic measurement. Furthermore, a 10-cm-thick foam (70 kg/m^3^, Jinniu, JSC, Linyi, China) was placed on the platform to modify the proprioceptive cues. Enhanced measurements of balance were implemented with eyes open or closed on the foam support (C3 and C4 respectively) [30]. Three trials were done in each test condition, and the mean value of postural sway denoted the final result. For consciously assessing subjects’ ability to adapt and regulate equilibrium appropriately to changed internal and external constraints, a mean equilibrium score (MES) was introduced by adding the mean individual condition scores and then dividing that sum by four [31].

### Radiographic and clinical assessments

The radiographic evaluations including several parameters like the mechanical medial proximal tibial angle (mMPTA), femorotibial angle (FTA), posterior tibial slope (PTS), and tibial posterior translation (TPT) of the affected knees, were measured one month pre- and one year postoperatively considering the bony healing time of different individuals after HTO. The clinical evaluation consisted of estimating the Western Ontario and McMaster Universities osteoarthritis index (WOMAC) [32], Lysholm knee score [33], visual analogue scale (VAS) [34], and the range of motion (ROM) of the affected knee after patients underwent posturography at each testing phase.

### Statistical analysis

The required sample size was calculated using PASS 2021 package software (NCSS, Kaysville, Utah, USA). A sample consisting of 23 patients and 23 controls was needed to obtain 90% power of study (1-Beta). Alpha was set to 0.05 for two-sided tests. The research data were analyzed using SPSS 22.0 software (IBM, Armonk, NY, USA). The normal distribution of quantitative data was checked by the Kolmogorov–Smirnov test. Qualitative data were displayed as numbers (n) and compared by Chi-square test. Postural sway in all conditions between three testing times and clinical evaluation items were compared by mixed-effect analysis of variance (normally distributed data) and then by Bonferroni correction for post hoc comparisons. Differences in postural stability between patients and control subjects were subsequently assessed by independent-samples t-test for normally distributed data. Radiographic evaluation parameters were assessed by paired sample t-test while linear regression analysis was used for correlating MES at each testing phase along with the radiographic and clinical outcomes. All statistically significant differences were defined as a probability level of *p* < 0.05. For significant within-group and between-group differences in postural sway, effect sizes were calculated using ŋ^2^ and Cohen’s d.

## Results

### Anthropometrics of participants

The anthropometrics characteristics of participants are summarized in Table 1. There were no significant differences in clinical variables between the study patients and control subjects (all *p* > 0.05).

**Table 1:** Characteristics of participants (mean ± SD)

Parameter	Study patients (*n* = 32)	Control subjects (*n* = 40)	Chi-square test *p*-value
Sex (*n*), male/female	20/12	24/16	0.325
Age (years)	55.2 ± 4.5	53.9 ± 3.6	0.639
Height (cm)	168.2 ± 6.4	169.4 ± 7.2	0.542
Weight (kg)	81.4 ± 7.3	78.1 ± 8.7	0.704
BMI (kg/m^2^)	28.9 ± 4.5	27.5 ± 3.9	0.298

### Variations in balance control in knee OA patients

**Fig. 1:**
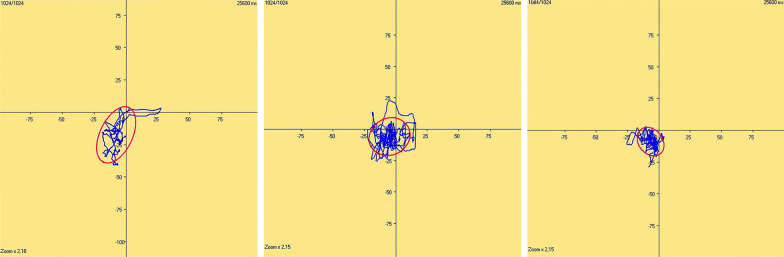
Schematic diagram of the changes in postural sway area (in mm^2^) covered by the center of foot pressure (CoP) trajectory 1 month before, 3 and 12 months after HTO from left to right

**Fig. 2:**
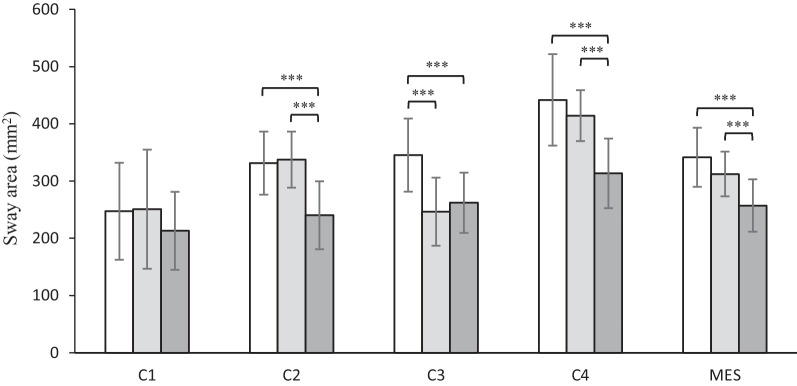
Mean values with standard deviations of sway area for four conditions (C1-C4) and mean equilibrium score (MES) in patients 1 month before (white bars), 3 (light gray bars) and 12 months after HTO (dark gray bars); ***p < 0.001

Figure 1 shows a schematic of the changes in postural sway area covered by the CoP trajectory before and after HTO in the same patient. The variations in balance control in OA patients in three testing phases are displayed in Fig. 2. There was no noticeable divergence of postural stability in C1 (*p* = 0.172, *ŋ*^2^ = 0.037). However, when adding disturbed information, significant heterogeneities were found in these patients, respectively, in C2 (*p* < 0.001, *ŋ*^2^ = 0.410), C3 (*p* < 0.001, *ŋ*^2^ = 0.354), C4 (*p* < 0.001, *ŋ*^2^ = 0.431) and MES levels (*p* < 0.001, *ŋ*^2^ = 0.377). The post hoc analysis showed that patients displayed a reduced sway area one year postoperatively (mean = 240.2 ± 57.8 mm^2^) compared to the preoperative C2 measurement (mean = 331.2 ± 54.6 mm^2^); a lower sway area (mean = 313.4 ± 61.3 mm^2^) was also observed one year postoperatively than the preoperative C4 measurement (mean = 441.7 ± 81.6 mm^2^). However, the sway area was not altered three months postoperatively (mean = 337.3 ± 49.7 mm^2^) compared to the C2 preoperative level. A similar outcome was also found three months postoperatively (mean = 414.1 ± 45.0 mm^2^) in contrast to the C4 preoperative level. In another testing condition evaluating the C3 measurement, patients displayed a lower sway area three (mean = 246.5 ± 59.8 mm^2^) and twelve months postoperatively (mean = 262.0 ± 53.0 mm^2^) compared to the preoperative value (mean = 345.3 ± 65.3 mm^2^), whereas no obvious alterations were found between the two postoperative tests.

The MES of postural sway was significantly improved one year postoperatively (mean = 257.2 ± 45.9 mm^2^) in contrast to that measured pre- (mean = 341.4 ± 50.9 mm^2^) and three months postoperatively (mean = 312.2 ± 39.2 mm^2^). Nevertheless, unchanged postural stability was observed three months postoperatively compared to its preoperative level.

### Differences in balance control between patients and control subjects

**Fig. 3:**
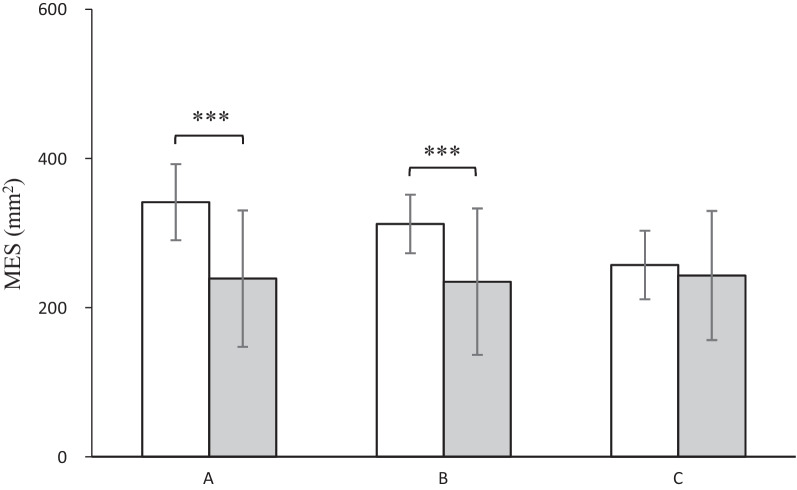
Mean values with standard deviations of sway area for mean equilibrium score (MES) in patients (white bars) and control subjects (gray bars) 1 month before (**A**), 3 (**B**) and 12 months after HTO (**C**); ***p < 0.001

Significant MES heterogeneities, which were calculated as a general reflection of balance control, were observed in patients and control subjects pre- (*p* < 0.001, Cohen’s *d* = 1.28) and three months postoperatively (*p* < 0.001, Cohen’s *d* = 0.99) (Fig. 3). Patients demonstrated a greater postural sway area before HTO (mean = 341.4 ± 50.9 mm^2^) than that of the control subjects (mean = 238.9 ± 91.5 mm^2^). Three months postoperatively, their postural sway area did not show a sharp decline (mean = 312.2 ± 39.2 mm^2^) compared to that of the control subjects (mean = 234.8 ± 98.2 mm^2^). However, comparable postural stability in patients was observed one year postoperatively (mean = 257.2 ± 45.9 mm^2^) in contrast to the healthy individuals (mean = 243.1 ± 86.4 mm^2^, *p* = 0.459, Cohen’s d = 0.17). However, no differences were observed between the three measurements of the control subjects.

### Radiographic and clinical evaluation outcomes

All the patients were observed a bone union during the postoperative follow-ups. The pre- and postoperative radiographic evaluations of the patients are shown in Table 2. Compared with the preoperative measurements, the affected knee joints showed significant increases in mMPTA (*p* < 0.001, Cohen’s *d* = 1.54) and PTS (*p* = 0.002, Cohen’s *d* = 0.23) values one year after osteotomy. Meanwhile, patients displayed a decreased FTA one year postoperatively in contrast to the preoperative value (*p* < 0.001, Cohen’s *d* = 2.32). Nevertheless, the pre- and postoperative TPT did not show a significant change (*p* = 0.069, Cohen’s *d* = 0.22).

**Table 2:** Radiographic evaluations of the patients before and after high tibial osteotomy (mean ± SD)

Parameter	1 month before HTO	1 year after HTO	*p*-value
mMPTA	81.9° ± 3.5°	88.2° ± 4.6°	< 0.001***
FTA	183.9° ± 3.1°	175.6° ± 4.0°	< 0.001***
PTS	10.6° ± 1.8°	11.0° ± 1.7°	0.002**
TPT	5.3 mm ± 1.0 mm	5.1 mm ± 0.8 mm	0.069

*HTO* high tibial osteotomy, *mMPTA* mechanical medial proximal tibial angle, *FTA* femorotibial angle, *PTS* posterior tibial slope, *TPT* tibial posterior translation

**p < 0.01, ***p < 0.001

**Fig. 4:**
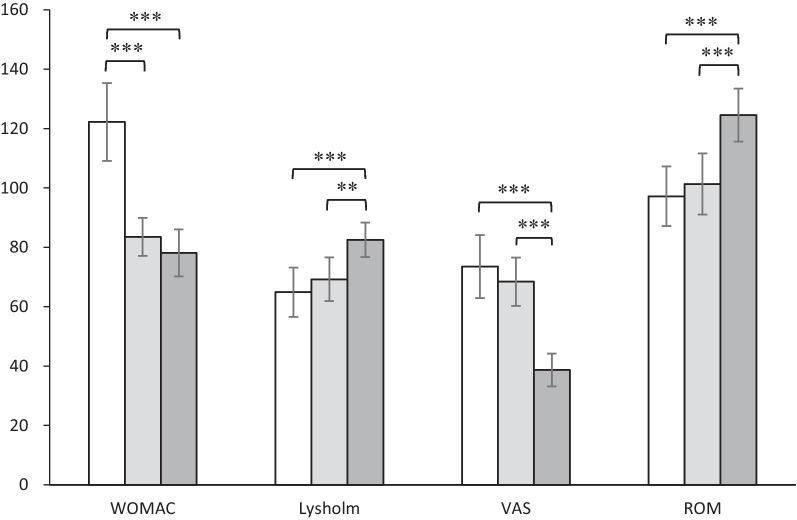
Mean values, associated with standard deviations, of clinical assessments of the affected knee joint observed in patients 1 month before HTO (white bars), 3 (light gray bars) and 12 months after HTO (dark gray bars); WOMAC: the Western Ontario and McMaster Universities osteoarthritis index; Lysholm: Lysholm knee score; VAS: visual analogue scale; ROM: range of motion; **p < 0.01, ***p < 0.001

The clinical assessment results of the affected knee joint in patients are shown in Fig. 4. Significantly decreased WOMAC index were observed three months (mean = 83.5 ± 6.4) and one year postoperatively (mean = 78.1 ± 7.9) than the preoperative value (mean = 122.2 ± 13.1) (*p* < 0.001, *ŋ*^2^ = 0.560). Improved Lysholm scores were also observed one year postoperatively (mean = 82.5 ± 5.8) in contrast to the pre- (mean = 64.9 ± 8.3) and three months postoperative values (mean = 69.3 ± 7.4) (*p* < 0.001, *ŋ*^2^ = 0.515). Remarkably decreased joint pain scores quantified by VAS were observed within one year following the surgical interventions (mean = 38.7 ± 5.5) compared to that evaluated pre- (mean = 73.5 ± 10.6) and three months (mean = 68.4 ± 8.1) postoperatively (*p* < 0.001, *ŋ*^2^ = 0.475). Moreover, an improved range of motion of the affected joint was noticed one year postoperatively (mean = 124.5° ± 8.9°) compared with the preoperative level (mean = 97.2° ± 10.1°) and that measured three months (mean = 101.3° ± 10.3°) after HTO (*p* < 0.001, *ŋ*^2^ = 0.602).

Table 3 displays the outcomes of Linear regression analysis correlating MES at each testing phase along with radiographic and clinical evaluations. Significant higher correlations were found between postural sway and WOMAC index (*p* = 0.001), VAS score (*p* = 0.021) and ROM (*p* = 0.034) as well as radiographic outcomes of mMPTA (*p* = 0.006) and PTS (*p* = 0.044) one year after HTO. Besides, the WOMAC index three months postoperatively also showed a significant association with the postural sway at that time (*p* = 0.026).

**Table 3:** Linear regression analysis comparing the mean equilibrium score at each testing phase along with the radiographic and clinical evaluations

	MES (pre-HTO)		MES (post-HTO_1_)		MES (post-HTO_2_)	
	*B*	*P*	*B*	*P*	*B*	*P*
WOMAC	1.307	0.113	2.505	0.026 *	3.307	0.001 **
Lysholm	−0.605	0.645	−0.844	0.375	−1.744	0.141
VAS	−0.484	0.687	0.487	0.573	1.940	0.021 *
ROM	1.227	0.250	0.083	0.897	−1.491	0.034 *
mMPTA	1.175	0.693	–	–	−3.937	0.006 **
FTA	3.482	0.361	–	–	1.533	0.323
PTS	3.424	0.531	–	–	6.602	0.044 *
TPT	4.688	0.633	–	–	1.770	0.797

*MES* mean equilibrium score, *pre-HTO* 1 month before high tibial osteotomy, *post-HTO*_*1*_ 3 months after high tibial osteotomy, *post-HTO*_*2*_ 12 months after high tibial osteotomy, *WOMAC* the Western Ontario and McMaster Universities osteoarthritis index, *Lysholm* the Lysholm knee score, *VAS* the visual analogue scale, *ROM* range of motion, *mMPTA* mechanical medial proximal tibial angle, *FTA* femorotibial angle, *PTS* posterior tibial slope, *TPT* tibial posterior translation, *B* unstandardized coefficient

*p < 0.05, **p < 0.01

## Discussion

This study demonstrated a significant improvement in balance control in patients with knee varus deformity after HTO; such patients displayed balance impairments preoperatively as compared to control subjects. After HTO, study patients displayed better postural stability in contrast to preoperative levels. Moreover, the restored quality of static balance might be comparable to that of healthy individuals.

Conventionally, balance orientation in an upright position requires visual and proprioceptive afferents to provide sensory information [35]. In our study, static bipedal balance control showed an undifferentiated performance pre- and postoperatively in patients under available visual and somatosensory afferent conditions that help in synergistically regulating a stable upright posture. Once appropriate and adequate sensory inputs are processed by the central nervous system, prompt and suitable responses are generated based on the available sensory signals, thus leading the subjects to a stable posture. In the absence of visual afferents, proprioception displays a predominance in maintaining balance and stabilizing body posture. A previous study demonstrated that vision plays an important role in maintaining postural stability as conduction and central integration become less efficient with age [36]. Our results displayed a significant improvement in balance orientation one year after HTO when patients closed their eyes than their preoperative level, suggesting more applicability of somatosensory inputs for postural control and a sensorimotor dominance shift from vision to proprioception. Furthermore, healthy individuals usually use more somatosensory information than vestibular and visual cues to perceive postural oscillation during a normal upright posture [37, 38]. Another study confirmed that proprioception and tactile sensation afferents from the lower extremity to the upper body play an essential role in maintaining balance and spatial orientation during quiet stance [39]. However, our results showed increased stable postural control performances after modification of proprioception postoperatively. When vision and somatosensory information were absent or interfered with, the subjects were restrained from producing adapted strategies due to an altered referable environment, thereby resulting in improper usage of sensory information to regulate body balance [31]. From our results, balance control was significantly improved 1 year postoperatively compared to preoperatively when the patient stood on the foam, both with eyes open and closed. This indicates an improved ability of the knee to maintain postural stability in a proprioceptive deficit environment 1 year after HTO. Interestingly, carefully comparing the difference between the two outcomes standing on foam with eyes open and closed, we found that in the open-eye condition, the patient's balance control improved more quickly, returning to a lower level 3 months postoperatively and maintained until 1 year postoperatively. In contrast, with the eyes closed, improved balance control was not observed until 1 year postoperatively. We believe this difference should be attributed to the synergistic role of vision in maintaining body balance. At 3 months postoperatively, the limited recovery of knee proprioception relies more on the synergistic effect of vision when the eyes are open to co-regulate good body balance. However, when vision is absent, the limited proprioceptive afferents are unable to provide sufficient sensory information to the central nervous system to maintain postural stability, such that the two different balance control performances occur. The improvement in MES regarding postural stability one year postoperatively reflects an altered sensorimotor integration, thus suggesting a restoration of efficient compensatory ability for maintaining balance.

A transverse comparison of patients and control subjects revealed obvious divergences in pre- and three-month postoperative findings. With the correction of lower extremity alignment, the balance control homogeneity between patients and healthy individuals was not observed until one year postoperatively. These findings are consistent with the results of the corrective osteotomy in patients with knee OA and valgus deformity as well as the UKA in unicompartment knee OA patients as mentioned above [15, 22]. Successful surgical interventions can significantly improve postoperative postural stability by restoring lower limb alignment. Meanwhile, clinical outcomes such as WOMAC index, pain score and range of joint motion are also improved. However, the difference from our study is that they used two osteotomies (distal femur or high tibial or double osteotomy) to correct valgus deformity while the patients with UKA were compared with their contralateral limb rather than the healthy controls. As another commonly used treatment for OA, TKA is proved effective at improving balance control six months after surgery, as well as all domains of quality of life. And yet, it is unable to restore the balance to a level comparable to that of healthy individuals [40]. Since several mechanoreceptors such as Pacinian corpuscles, Ruffini endings and Golgi organs are present within articular cartilage [41], multiple lines of evidence suggest that lower extremity proprioception is altered with the degeneration of intra-articular structures, and these alterations include the mechanisms involving decreases in the acuity, sensitivity, and integration of the proprioceptive signals [42, 43]. Therefore, we speculate that the postoperative somatosensory deficit that can result from the replacement of degenerative cartilage, cruciate ligaments as well as menisci by a prosthesis in these TKA patients may be responsible for the limited restoration of balance control. Generally, for most of the patients undergoing an osteotomy, a preliminary bone union was achieved within three months postoperatively. In our study, although decreased postural oscillation was observed three months postoperatively, the quality of balance control in the patients is still not able to return to normal. Based on the results of our clinical evaluation, we believe that the limited improvement in joint pain and range of motion three months after surgery may lead to inefficient usage of anticipatory strategies and generation of compensatory movements to maintain postural equilibrium, even if HTO preserves as much proprioception of the knee as possible.

The knee joint plays a compensatory role in the regulation of postural stability in a quiet stance, and allows the lower limb to consistently track the center of the motion [35]. According to our radiographic findings, a varus-producing osteotomy may optimally correct lower limb alignment by improving mMPTA and FTA as well as relieve cartilage loading force, and shift the stress from the medial to the lateral compartment, thereby redistributing barycenter in the coronal plane. Meanwhile, corrected PTS decreases the tibial anterior compressive strains in the sagittal plane to ensure the postoperative knee stability, thus improving the patients’ balance control. On the other hand, decreased WOMAC index and pain scores, increased Lysholm scores and range of joint motion after HTO indicate a recovery of knee function that is highly conducive to the restoration of balance control. The pain has been proved to be an important predictor of postural sway in knee OA patients [44]. Moreover, normal contraction and straining of lower limb muscles could functionally enhance joint integrity by improving mechanical restraints and mediate knee stability through reflexive muscle responses provoked by mechanoreceptors originating in the periarticular structures [45]. Hence, individuals with medial knee OA display increased compensatory co-contraction of lower limb muscle and excessive joint laxity during walking [46, 47]. Before conducting HTO in our study, periarticular muscles, such as quadriceps femoris, hamstring, and gastrocnemius, regulated body equilibrium by enhancing antagonistic co-contraction and mutually collaborating under malaligned conditions, especially in a sensory deficit environment. When lower limb alignment is rectified by HTO, the normal transmission of strain reduces the compensatory load of muscles and generates faster and more efficient responses via decreased muscle co-contraction and adequate sensorimotor strategies to mediate postural stability. Besides, the preserved mechanoreceptors of the synovial joint, Golgi tendon organs and muscle spindles provide necessary sensory information for recovering joint function in patients postoperatively. Overall, the restoration of lower extremity alignment reduces the affected compartment’s load and mitigates the damage of mechanoreceptors in the cartilage. Accordingly, we suggest that the effect of HTO on proprioception may be attributed to improved mechanoreceptor perception of stress transmission, joint mobility, muscle contraction and pain signal transmission, thus providing more accurate sensory information than preoperatively, allowing the body to produce faster and more efficient responses through feedback and coordination of the central nervous system, and improving somatic balance control under basic and complex conditions and efficiency of the entire sensorimotor chain, ensuring a stable anticipatory and compensatory balance strategy. Nevertheless, even if the revival of balancing ability seems inadequate within three months after HTO, it is suggested that physical function renewal occurs much later, optimally one year post-surgery [22, 48].

There were several limitations to this study. Although we had excluded patients with bilateral knee varus deformity and selected unilateral knee varus patients as subjects to avoid the bilateral osteotomy bias, the contralateral knees had occasional osteoarthritis, which could have affected balance control postoperatively. Furthermore, frequent equilibrium tests should be conducted between three months and one year postoperatively to assess the changes in dynamic balance responses. Future studies should be consequently undertaken that include the conditions of both the knees and frequent balance tests during the postoperative convalescence.

## Conclusions

In conclusion, this study showed that HTO improves balance control in patients with knee OA and a varus deformity and enhances the likelihood of normal postural stability. Improved postural performance could be relevant to the correction of lower limb alignment and the improvement of joint function, thus leading to a recovery of sensorimotor efficiency and restoration of anticipatory and compensatory strategies implementing balance control. This reacquisition also allows the knee joint to recover its corrective compensatory role in postural regulation and should be considered while deciding the management of patients with knee OA and lower extremity malalignment.

## Data Availability

All data generated and analyzed during this study are available from the corresponding author on reasonable request.

## Abbreviations

OA: Osteoarthritis
HTO: High tibial osteotomy
TKA: Total knee arthroplasty
UKA: Unicondylar knee arthroplasty
MRI: Magnetic resonance imaging
CoP: Center of foot pressure
MES: Mean equilibrium score
mMPTA: Mechanical medial proximal tibial angle
FTA: Femorotibial angle
PTS: Posterior tibial slope
TPT: Tibial posterior translation
WOMAC: Western Ontario and McMaster Universities osteoarthritis index
VAS: Visual analogue scale
ROM: Range of motion
